# PID15, a novel 6 kDa secreted peptide, mediates *Naja naja* venom phospholipase A_2_ induced apoptosis in isolated human peripheral lymphocytes

**DOI:** 10.1186/s12929-014-0066-2

**Published:** 2014-07-17

**Authors:** Mukunda Chethankumar, Leela Srinivas

**Affiliations:** 1 Postgraduate Department of Biochemistry, JSS College of Arts, Commerce and Science (Autonomous), Ooty Road, Mysore 570 025, Karnataka, India; 2 Adichunchanagiri Biotechnology and Cancer Research Institute, Balagangadharanatha Nagara–571 448, Nagamangala Taluk, Mandya, Karnataka, India

**Keywords:** PLA2, Apoptosis, DNA fragmentation, Naja naja venom, Lymphocytes, PID15

## Abstract

**Background:**

Snake venoms are a complex mixture of active principles mainly peptides and proteins also including amino acids, nucleotides, free lipids, carbohydrates and metallic elements bound to proteins that interfere in several biological systems. In this study, we aimed to understand the mode of action of the apoptosis inducing ability of *Naja naja* venom phospholipase A_2_ (NV-PLA_2_) using isolated human peripheral lymphocytes.

**Results:**

Human peripheral lymphocytes when incubated with *Naja naja* venom phospholipase A_2_ (NV-PLA_2_) induced up to 68% DNA fragmentation. The dialysed conditioned media obtained by incubating lymphocytes with NV-PLA_2_ at 15^th^ min induced 44% DNA fragmentation, referred to as cmlp-active. Cmlp-active showed 20.5% increased protein concentration than the corresponding control condition media cmlp-c-15. Test for creatine kinase activity in cmlp-active proved negative and negligible amount of lactate dehydrogenase did not show significant DNA fragmentation. Fractionation of cmlp-active on Sephadex G-25 showed two peaks, major peak induced 38% DNA fragmentation, which was further rechromatographed on Sephadex G-25. The single peak obtained was named PID15 (P*hospholipase A*_*2*_*Induced* D*NA fragmentation factor secreted at* 15^*th*^ min). Q-Tof MS/MS analysis of PID-15 showed it is a 6 kDa peptide. PID15 sequence analysis gave 40 amino acids in the following order, msilpcknvs iwvikdtaas dkevvlgsdr aikflylatg. The homology search for the sequence revealed it to be an Apoptosis Inducing Factor (AIF).

**Conclusion:**

Results indicate that the secretion of PID15 is dependent on concentration of NV-PLA_2_ treatment, incubation time and also on temperature and the probable membrane origin of PID15 and not of cytosolic origin with apoptosis inducing ability.

## Background

Snake bite envenomations constitute public health hazards in many regions of the world, particularly in the tropics [[1]–[4]]. Approximately 450 of the 3000 species of snakes found worldwide are considered to be dangerous to humans. Of these about 52 are encountered in the geographical area, which extends from Pakistan and the rest of the Indian subcontinent through to the Philippines and Indonesia. In many parts of this region, snake bite is a familiar occupational hazard of farmers, plantation workers and others, resulting in tens of thousands of deaths each year and innumerable cases of chronic physical handicap. An estimated 55000 to 60000 mortalities are reported in India from bites of venomous snakes [[5]].

Snake venoms are a complex mixture of active principles mainly peptides and proteins also including amino acids, nucleotides, free lipids, carbohydrates and metallic elements bound to proteins that interfere in several biological systems [[6],[7]]. Among the enzymic factors, phospholipase A_2_ (PLA_2_) has been identified as important group of enzymes in inducing pharmacological effects [[8]]. Snake venom PLA_2_s among the smallest enzymes (~13-15 kDa), are highly stable (due to the presence of 5–8 disulfides), and have a conserved structural scaffold [[9]]. Furthermore, PLA_2_s can be monomeric, homomultimeric or heteromultimeric. Finally, a single snake venom can contain a variety of PLA_2_s (up to 15 distinct enzymes), and the genes for these PLA_2_s are likely to have evolved through positive Darwinian selection [[10]]. Symptoms of snake envenomation are a combination of the complex protein and non protein components present in the venom. These effects at later stages result in tissue damage, which reaches many organs, such as the brain, lung, kidney, heart, and liver [[11]–[13]].

Clinically, the most common and important effect of cobra envenomation is local tissue necrosis [[14]]. Victims may suffer extensive muscle damage leading to rhabdomyolysis and the loss of muscle-specific protein [[15]]. Snake venom induced muscle necrosis is attributed to single chain peptides consisting of 42–44 amino acid residues and PLA_2_s representing either single chain proteins or existing as complexes of several enzyme subunits or combined with other non enzymatic proteins [[16]]. Local tissue necrosis causes local pain and swelling, and may be associated with blistering at the bite site. Venom induces cell death by two important pathways, either by necrosis or apoptosis.

Cell death by necrosis is attributed to H_2_O_2_ produced by oxidation of alpha-amino acids [[17]]. At a later stage, when H_2_O_2_ is scavenged by the action of catalase, a switch to apoptosis takes place. Venom PLA_2_s are able to induce apoptosis through calpain mediated activation of caspases and other pro apoptotic proteins, and also by formation of permeability transition pore in the membrane as a result of increased Ca^2+^ in the mitochondrial matrix [[18]]. They are also known to induce apoptosis via a mechanism which involve ceramide generation. These lipid signaling molecules are generated by the direct effect of hydrolysis of membrane phospholipids [[19]]. However, it is through the necrosis, cells could go into apoptosis through the mechanisms mentioned above.

Necrosis induces plasma membrane rupture, release of cytosolic components, prominent Ca^2+^ influx, mitochondrial Ca^2+^ overload and pycnotic nuclei [[15]]. Hence, understanding cell death as a consequence of necrosis is of great importance in preventing cell death and local tissue damage. Despite the great advances in the understanding of morphological and biochemical alterations associated with cell injury, the apoptosis inducing effects of *Naja naja* (Indian Cobra) venom PLA_2_s at the cellular level has not been well characterized. In this study, we attempt to clarify the mode of action of the apoptosis inducing ability of *Naja naja* venom phospholipase A_2_ (NV-PLA_2_) using isolated human peripheral lymphocytes.

## Methods

### Materials

Spectrapar dialysis membrane *(2 kDa, 8 kDa and 12 kDa MWCO)*, CM-Sephadex C25, Sephadex G25, Sephadex G10 and Sephadex G50, Hind III digest of λ-phage DNA were purchased from Sigma Aldrich Co., USA. Agarose, Bovine Serum Albumin (BSA), trifluoro acetic acid (TFA), acetonitrile, dilinoleoyl phosphatidyl choline (DL-PC), lipoxygenase, deoxycholate, NaCl, KCl, MgSO_4_, D-Glucose, EDTA, Diphenyl amine (DPA) were purchased from E-Merck India. *N. naja* venom was obtained from Haffkine Institute for Training, Research and Testing, Mumbai, India. The lyophilized powdered venom was dissolved in 0.9% saline (0.1 mg/ml) and preserved at 4°C until use. All other chemicals and reagents used were of analytical grade and organic solvents were distilled prior to use.

### Purification of PLA_2_ from Naja *naja* venom

Purification of PLA_2_ from *Naja naja* venom was essentially followed according to the method described [[20]] with minor modifications. *Naja naja* venom (600 mg, equivalent to 580 mg of protein) was dissolved in 20 mM phosphate buffer, pH7.0 and fractionated on a CM-Sephadex C-25 column (1.6×135 cm). The fractions were eluted in a step wise manner using phosphate buffer of different molarities (0.02–0.3 M) and different pH values (7.0–8.0). The fractions were collected at the flow rate of 1.5 ml for 5 min. The protein elution was monitored at 280 nm using UV-1601A Shimadzu spectrophotometer. The fractionation was carried out at 4°C. Fractions eluted were assayed for the enzyme activity. PLA_2_ activity was determined by mean of a coupled assay using dilinoleoyl phosphatidyl choline (DL-PC) as a substrate and lipoxygenase as coupling enzyme [[21]]. The fractions with enzyme activity was pooled, desalted, lyophilized and stored at −20°C. Fractions eluted with 0.22 M phosphate buffer, pH8.0 showed was designated as peak XI.

### Rechromatography of peak XI on CM-Sephadex C-25 column

Peak XI (62 mg in 2 ml of 0.02 M phosphate buffer, pH7.0) was rechromatographed on CM-Sephadex C-25 column (1.4×75 cm), pre-equilibrated with 0.02 M phosphate buffer, pH7.0. Protein was eluted in a step wise manner using phosphate buffer of different molarities (0.02–0.15 M) and pH (7.0–8.0) values. The fractions were collected with a flow rate of 2.5 ml for 5 min at 4°C. The eluted fractions were monitored at 280 nm. Each fraction was assayed for PLA_2_ activity [[20]] and the major peak – peak III (31 mg in 1 ml of 0.1 M NaCl) recovered was chromatographed on Sephadex G-50 column (1.0×90 cm), pre-equilibrated with 0.1 M NaCl. Protein was eluted with a flow rate of 1.0 ml for 5 min at 4°C. The eluted fractions were monitored at 280 nm. The major peak with PLA_2_ activity was purified on reversed phase column using Waters C_18_ μBondpak reverse phase column (7.8 mm×300 mm), bead size 10 μm and porosity 125 Å) pre-equilibrated with 0.1% trifluoro acetic acid (TFA) in water. About 50 μg of protein, pre-incubated with 0.1% TFA was applied on to the column. Protein was eluted with 0-90% water:acetonitrile gradient at a flow rate of 1 ml/min for 70 min. Protein elution was monitored at 280 nm. Single peak obtained was designated as NV-PLA_2_ (*Naja naja* venom phospholipase A_2_).

### Treatment of lymphocytes with NV-PLA_2_ and preparation of conditioned media

The peripheral lymphocytes were isolated from 10 to 15 ml of freshly drawn venous blood from healthy male donors aged between 25–30 years [[22]]. Condition media was prepared according to the method described by [[22],[23]] with slight modifications. Lymphocytes (1×10^6^ cells/ml) were incubated with NV-PLA_2_ (10 μg) in a total reaction mixture of 5 ml of Hank’s Balanced Salt Solution, pH7.4 (HBSS, 137 mM NaCl, 5 mM KCl, 8.5 mM phosphate buffer, pH7.4, 0.8 mM MgSO_4_ and 5 mM D-glucose) at 37°C for varying time intervals from 0 min to 30 min with 5 min time interval in culture flasks. At the end of the incubation period, the reaction was arrested by placing the reaction flasks in ice bath. The cells were then centrifuged at 1200×*g* for 8 min at 4°C. The supernatant was collected and referred as Conditioned Media (CM). The CM thus obtained at 0 min, 5 min, 10 min, 15 min, 20 min, 25 min and 30 min time periods were referred to as cmlp-0, cmlp-5, cmlp-10, cmlp-15, cmlp-20, cmlp-25 and cmlp-30 respectively.

### Conditioned media induced DNA fragmentation

Each of the Conditioned Media (CM) obtained at each time point from cmlp-0 to cmlp-30 was dialysed through spectrapar dialysis membranes of cutoff 2Kd, 8Kd and 12Kd separately for 72 hrs against 10 mM phosphate buffer, pH7.4 with 6 changes at an interval of 12 hrs. Similar experiment was carried where in, NV-PLA_2_ (10 μg) was added to the lymphocytes (1×10^6^ cells/ml) in a total reaction mixture of 5 ml of HBSS at the end of the incubation period. This served as control conditioned media. The dialysate obtained with 2Kd, 8Kd and 12Kd cutoff membranes were added to fresh batch of isolated lymphocytes and tested for DNA fragmentation [[24]]. The dialysate obtained with 2Kd cutoff membrane at 15 min was quantitatively recovered with minimum amount of HBSS, pH7.4 and was referred to as cmlp-active. The concentrations and dilutions of NV-PLA_2_ used were not lethal to the cells.

### Purification of cmlp-active

The cmlp-active was purified with the combination of gel permeation chromatography and *rp*-HPLC. 2200 μg of total protein of cmlp-active was loaded on to Sephadex G-25 column *(Vt = 60 ml, V0 = 20 ml)* and eluted with double distilled water. The fractions were collected at the flow rate of 1.0 ml/5 min. The eluted fractions were monitored at 280 nm in Shimadzu Spectrophotometer. Two peaks obtained were pooled separately, lyophilized and the DNA fragmentation was quantified for each peak [[24]]. The fractions of peak II showing maximum DNA fragmentation was refractionated on Sephadex G-25 column. 1000 μg of protein of peak II was loaded on to Sephadex G-25 column *(Vt = 60 ml, V0 = 20 ml)* and eluted with double distilled water. The fractions were collected at the flow rate of 1.0 ml/5 min. The eluted fractions were monitored at 280 nm in Shimadzu Spectrophotometer. A single peak was obtained called as (P*hospholipase A*_*2*_ I*nduced* D*NA fragmentation factor released at* 15^*th*^*min*) PID15. The homogeneity of this peak was tested on *rp*-HPLC and molecular mass of PID15 was determined using Hewlett–Packard (Model HP-1100).

Electrospray ionization was carried out using a capillary with an ID of 0.1 mm. The tip was held at 5000 V in a positive ion detection mode. Nebulization was assisted by N_2_ gas (99.8%) at a flow rate of 10 l/min. the spray chamber was held at 300°C. Data was acquired over a suitable mass range using a conventional quadrupole detector with cycle time of 3 s. The purified factor PID-15 was subjected to Ninhydrin test to check the presence of amino acids. It was tested for sugars by phenol-sulphuric acid method [[25]]. It was also tested for the presence of SH and − S − S − groups using nitroprusside [[26]] and for the presence of cysteine/cystine by Ellman’s method [[27]].

### Kinetics on the secretion of PID15

Lymphocytes were divided into three batches. In batch I, cells were incubated with different dose of NV-PLA_2_ (2 μg–14 μg) for 15 min at 37°C. In batch II, cells were incubated with NV-PLA_2_ (10 μg) for different time periods starting from 0 min to 30 min at 37°C. In batch III, cells were incubated with NV-PLA_2_ (10 μg) for 15 min at different temperature ranging from 20°C to 60°C with an interval of 10°C. The experiments were carried out with appropriate controls. After the incubation period, cells were processed and the DNA fragmentation was quantified.

### Effect of exocytosis inhibitors and antioxidants on the secretion of PID15

To demonstrate the possible mode of release of the factor, either from cytoplasm or from the membrane, several exocytosis inhibitors were used. Among the inhibitors, 200 μM Quin-2 AM (Intracellular Ca^2+^ chelator), 500 μM EGTA (extracellular Ca^2+^ chelator) and 12 μg/ml Cytochalasin B (microfilament formation inhibitor) were used at the concentrations which were not lethal to the cells as mentioned in the literature [[28],[29]]. The effect of Superoxide dismutase (0.6 U/μl), Catalase (0.2 U/μl) and Turmerin (180 nM) on the secretion of PID15 was tested. The experiments were carried out with appropriate controls. The doses used were in compliance with the reported literature.

### Sequence analysis of PID15

PID15 was subjected to peptide sequence analysis using LCMS. PID15 was first subjected to trypsin digestion and then the fragments obtained were analyzed and the order of the fragments was also determined. The sequence obtained was further analyzed for sequence homology and other parameters using the online proteomics tool, ExPasy. Various parameters such as molecular mass, stability and extinction coefficient were determined.

### Statistical analysis

Statistical analysis was done in SPSS (Windows Version 10.0.1 Software Inc. New York) using a one-sided students *t-*test. All the values represent mean of triplicates and are expressed as Mean ± SD. *p* < 0.01, *p* < 0.05 was considered as significant and *p* < 0.001 was considered as highly significant.

## Results and discussion

The cell mediated cytotoxicity may be dependent on transmembrane signaling or exocytosis of toxic materials such as perforin and proteases by the killer cells [[30]]. Secretion of perforin and proteases is dependent on extracellular calcium. Mitochondria play a crucial role in regulating cell death, which is mediated by outer membrane permeabilization in response to death triggers such as DNA damage and growth factor deprivation [[31]]. As a consequence, multiple death-promoting factors residing in the mitochondrial intermembrane space are liberated in the cytosol. Once released into the cytosol, these mitochondrial proteins such as Cytochrome C and Apoptosis Inducing Factors (AIFs) activate both caspase-dependent and caspase-independent cell death pathways [[32],[33]].

Snake venom components are known to induce cell death through many mechanisms and one such process is apoptosis. Earlier studies have shown that a cytotoxic substance from Korean snake venom which is responsible for the apoptosis is L-amino acid oxidase, a glycoprotein of 110 kDa. This protein generates H_2_O_2_ by catalyzing oxidation of L-amino acid [[34]]. Another study show that OHAP-1, a Okinawa Habu Apoxin Protein-1 isolated from Okinawa Habu *(Trimeresurus flavoviridis)* venom induce apoptosis, mediated through promoting generation of intracellular reactive oxygen species (ROS) [[35]]. Myotoxic PLA_2_s isolated from the snake venoms have shown to induce necrosis [[36]], in which the disruption of plasma membrane lead to collapse of ionic gradients with an increased Ca^2+^ influx. This process could further lead to apoptosis through calpain mediated activation of caspases and other pro apoptotic proteins, and also by formation of permeability transition pore in the membrane [[18]]. These PLA_2_s are also known to induce apoptosis via a mechanism which involve ceramide generation [[19]].

Previous studies on primary human embryonic kidney (293 T) and mouse myoblast (C2C12) cell lines incubated with Egyptian cobra *(Naja haje)* venom, showed that the culture media contained enzymes such as Creatine Kinase (CK) and Lactate Dehydrogenase (LDH). These enzymes are used as marker for in vitro cytotoxicity. Release of CK and LDH enzymes into the culture media induced by the venom correlated well with morphological changes such as necrosis and apoptosis and the extent of cell death [[37]]. Despite the above understanding on cytotoxic and apoptosis inducing effect of snake venoms, there is no information on how the cells respond to the snake venom PLA_2_s and by which way the cells undergo apoptosis.

In this context, the present investigation was focused in understanding the apoptosis inducing effect of NV-PLA_2_ using isolated human peripheral lymphocytes. An attempt is also made to know whether the cells upon incubation with NV-PLA_2_ do release any cell death inducing factor(s) and changes that occur in the intracellular environments and also to understand the mode of release of such factor(s).

### Purification of PLA_2_ from *Naja naja* venom

**Figure 1:**
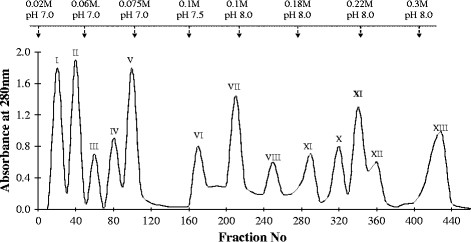
**CM Sephadex C-25 column chromatography of** ***Naja. naja*** **venom.** Elution profile of *N. naja* venom, 600 mg of *Naja. naja* venom, equivalent to 580 mg of protein, in 20 mM phosphate buffer, pH7.0 fractionated on CM-Sephadex C-25 column (1.6×135 cm), fractions eluted in step wise manner using phosphate buffer of different molarities (0.02–0.3 M) and pH values (7.0–8.0). Fractions collected at flow rate of 1.5 ml/5 min, monitored at 280 nm. Peak XI chosen for further purification.

*Naja naja* venom (600 mg) on CM-Sephadex C-25 column yielded 13 peaks. All the 13 peaks obtained did not show PLA_2_ enzyme activity as tested against dilinoleoyl phosphatidyl choline (DL-PC) substrate. Peak I, II, III, IV, VII, IX and XI showed PLA_2_ enzyme activity. Peak XI, eluted with phosphate buffer, 0.22 M, pH8.0 was the major peak with PLA_2_ activity with 22% protein yield when compared to the whole venom (Figure 1).

### Rechromatography of peak XI on CM-Sephadex C-25 column

**Figure 2:**
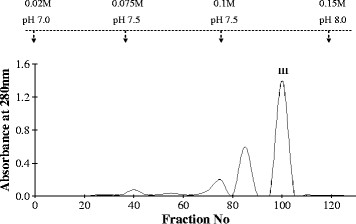
**Rechromatography of peak XI on CM Sephadex C-25 column.** Peak XI (62 mg in 2 ml of 0.02 M phosphate buffer, pH7.0) rechromatographed on CM-Sephadex C-25 column (1.4×75 cm), pre-equilibrated with 0.02 M phosphate buffer, pH 7.0. Protein eluted in phosphate buffer of 0.02–0.15 M and pH 7.0–8.0. Fractions collected at 2.5 ml/5 min at 4°C, monitored at 280 nm. Peak III chosen for further purification.

In the next step, 62 mg protein of peak XI was rechromatographed on CM-Sephadex C-25 column (Figure 2) and eluted with varying molar concentrations of phosphate buffer (0.02–0.15 M) and pH (7.0–8.0) values. The elution profile showed three peaks with peak III being the major one having PLA_2_ activity. This peak got eluted at 0.1 M phosphate buffer of pH7.5. The *rp-*HPLC of NV-PLA_2_ showed a single peak with retention time at 15^th^ minute. The molecular weight of NV-PLA_2_ as determined by SDS-PAGE was ~13 kDa (data not shown). The NV-PLA_2_ thus obtained is different from the PLA_2_s reported earlier from the same venom. A neurotoxic PLA_2_ variant isolated from *Naja naja* venom showed a relative molecular weight of 10 kDa [[3]]. A lethal PLA_2_ (NN-IVb1-PLA_2_) from *Naja naja* venom has been reported with pl value between 7–7.5 and molecular weight between 11,000-11,500. The PLA_2_ is devoid of myotoxic, anticoagulant, edema inducing and direct hemolytic activities [[38]].

### Conditioned media induced DNA fragmentation

Human peripheral lymphocytes when incubated with NV-PLA_2_ induced up to 68% DNA fragmentation. The DNA damage induced by conditioned media obtained at different time intervals and membrane cutoff is shown in Table 1. The conditioned media obtained at 15^th^min when dialysed with 2 kDa membrane cutoff induced up to 44% DNA fragmentation. This was referred to as cmlp-active. In a similar experiment, the conditioned media was obtained by treating lymphocytes with NV-PLA_2_ after each incubation period, which is thought to contain no DNA fragmentation and or cell death inducing factor(s). The control conditioned media showed only 7 to 9% DNA damage at 15^th^ min with 2 kDa membrane cutoff. The DNA fragmentation induced by cmlp-active was statistically significant when compared to its respective control conditioned media (cmlp-c-15). 12-*O-*Tetradecanoyl Phorbol Acetate (TPA) at 160 nM concentration was used as positive control, which induced up to 47% DNA damage in 15 min. The viability of cells at all stage was >90%. The cmlp-active showed 1790 μg of total proteins [[39]] whereas the corresponding cmlp-c-15 showed only 1485 μg of total protein. There was an increase in total protein concentration by 20.5%, which was highly significant. The UV spectral analysis of cmlp-active between 200–400 nm showed a peak at 220 nm indicating the peptide nature. There was no CK activity. There was negligible amount of LDH, which did not show significant DNA fragmentation.

**Table 1:** DNA damaging activity of the dialysed conditioned media

**Conditioned media (CM)**	**% DNA fragmentation**
Cmlp – 0 min	
12 kDa dialysate	10 ± 1.24
8 kDa dialysate	9 ± 1.01
2 kDa dialysate	11 ± 1.89
Cmlp – 5 min	
12 kDa dialysate	8 ± 0.89
8 kDa dialysate	9 ± 0.92
2 kDa dialysate	11 ± 1.97
Cmlp – 10 min	
12 kDa dialysate	8 ± 0.78
8 kDa dialysate	9 ± 0.85
2 kDa dialysate	27 ± 2.75
Cmlp – 15 min	
12 kDa dialysate	9 ± 1.11
8 kDa dialysate	18 ± 1.96
2 kDa dialysate	44 ± 3.26^@^
Cmlp – 20 min	
12 kDa dialysate	10 ± 1.54
8 kDa dialysate	8 ± 0.76
2 kDa dialysate	15 ± 1.41

The conditioned media obtained at 0 min, 5 min, 10 min, 15 min, 20 min, 25 min and 30 min time periods, referred as cmlp-0, cmlp-5, cmlp-10, cmlp-15 and cmlp-20 respectively, dialysed using membranes of cutoff 2Kd, 8Kd and 12Kd. Dialysate added to fresh normal lymphocytes (1×10^6^) cells, tested for DNA damage. cmlp (conditioned media obtained from lymphocytes treated with NV-PLA_2_), kDa (kilo Dalton), SD (Standard Deviation).

^@^*p* < 0.05 compared to corresponding control conditioned media (cmlp-c-15) as mentioned in Methods. The values expressed are mean of five experiments and are expressed as Mean ± SD.

### Purification of cmlp-active

**Figure 3:**
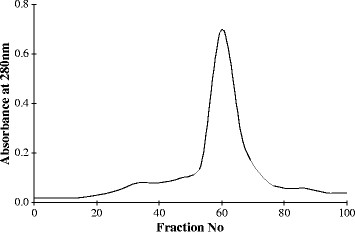
**Re-chromatography of peak II on Sephadex G-25 column.** Refractionation of peak II on Sephadex G-25 column, 1000 μg of protein of peak II loaded on Sephadex G-25 column *(Vt = 60 ml, V0 = 20 ml)* and eluted with double distilled water. Fractions collected at flow rate of 1.0 ml/5 min. A single peak was obtained called as (P*hospholipase A*_*2*_ I*nduced* D*NA fragmentation factor released at* 15^*th*^*min*) PID-15.

**Figure 4:**
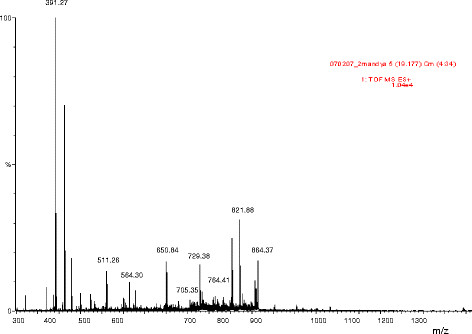
**ESI–LCMS of PID15.** Electro spray ionization spectra of PID15 recorded on a Hewlett – Packard (Model HP-1100) electrospray mass spectrometry. Data was acquired over a suitable mass range using a conventional quadrupole detector with cycle time of 3 s.

The cmlp-active was further fractioned and the major peak (peak II) obtained induced 38% DNA fragmentation when compared to peak I, which induced only 11% DNA fragmentation. Peak II was further rechromatographed on Sephadex G-25 and single major peak obtained induced up to 37.2% DNA fragmentation. This peak was referred as PID15 (P*hospholipase A*_*2*_ I*nduced* D*NA fragmentation factor released at* 15^*th*^*min*). The *rp-*HPLC of PID15 showed single peak with a retention time at 27^th^ min (Figure 3), indicating the purity of the protein PID15 isolated. Chemical characterization of PID15 showed negative to sugar residues indicating the non-glycoprotein nature of PID15. Q-Tof MSMS analysis of PID15 showed it is a small peptide with the molecular mass of 6 kDa (Figure 4).

There are few reports on lethal/toxic proteins in snake venoms which mediate apoptosis. BotroxLAAO is a glycoprotein with 12% sugar and an acidic character, as confirmed by its amino acid composition, rich in “Asp and Glu” residues. It displays high specificity toward hydrophobic l-amino acids revealed a significant increase in the apoptotic index of several cancer cell lines such as HL60, Jurkat, B16F10 and PC12 [[40]]. Acurhagin-C, an ECD disintegrin, a member of versatile metalloproteinase disintegrins from *Agkistrodon acutus* venom, has been identified as a platelet aggregation inhibitor, is shown to inhibit integrin alphavbeta3-mediated human endothelial cell functions by inducing apoptosis via caspase-3 activation [[41]]. BaP1, a snake venom metalloproteinase induce apoptosis in a non-adherent cell line, is likely to be related to its ability to induce cell cycle arrest. Processes other than apoptosis could be also involved in the cell death mechanism mediated by BaP1 on BAEC and HeLa cell lines [[42]]. However, molecular studies in inducing apoptosis by snake venom PLA_2_s are limited and there are no reports on how the cells undergo apoptosis following snake envenomations.

### Kinetics on the secretion of PID15

The optimum concentration of NV-PLA_2_ for the secretion of PID15 was 10 μg, above this concentration there was no significant release of PID15. Studies on time course showed that at 15^th^ min there was the secretion and above this time there was no secretion of PID15. The optimum temperature for the secretion of PID15 was at 37°C. Above this temperature there was no release of PID15. The above results thus indicate that the secretion of PID15 is dependent on concentration of NV-PLA_2_ treatment, incubation time and also on temperature.

### Effect of exocytosis inhibitors and antioxidants on the secretion of PID15

Exocytosis inhibitors such as 200 μM Quin-2 AM (Intracellular Ca^2+^ chelator), 500 μM EGTA (extracellular Ca^2+^ chelator) and 12 μg/ml Cytochalasin B (microfilament formation inhibitor) had not effect on the DNA fragmentation and also on the secretion of PID15. Quin-2 AM demonstrates a strong affinity for calcium. Cytochalasin B is a cell-permeable mycotoxin, inhibits cytoplasmic division by blocking the formation of contractile microfilaments. EDTA, a polyamino carboxylic acid is a strong calcium chelator. The secretion and apoptosis inducing effect of PID15 is not dependent on intracellular calcium. Apoptosis can be triggered by two major pathways, the death receptor-associated extrinsic pathway and the mitochondria-dependent intrinsic pathway. Caspases, a family of cysteine aspartyl-specific proteases, regulate the initiation and the final execution of apoptosis in both pathways. During cell injury there is significant increase in the level of intracellular calcium thus activating calpain, a non specific protease, which in turn activate caspase-12. The caspase-12 is then translocated to membrane activating caspase-3 inducing apoptosis. Thus, stating the probable membrane origin of PID15 and not from cytosol.

Earlier study has reported that there is an association between PLA_2_ and albumin (Ref. as below). Serum proteins, in particular albumin, provide a protective ‘buffer’ against small increases in PLA_2_ activity. Since enzymatic activity of NV-PLA_2_ is much critical in exhibiting the lethal effect, albumin if present in the culture medium does not affect the enzyme activity and further do not affect the apoptotic inducing activity of NV-PLA_2_ through secretion of PID15. The secretion of PID15 is of membrane origin and hence hydrolysis of membrane phospholipids occurs due to the enzymatic activity of NV-PLA_2_. It is understood that apoptotic inducing activity of NV-PLA_2_ is not affected by presence of albumin and it still exists in serum, a condition reflective to blood circulation [[43]].

**Figure 5:**
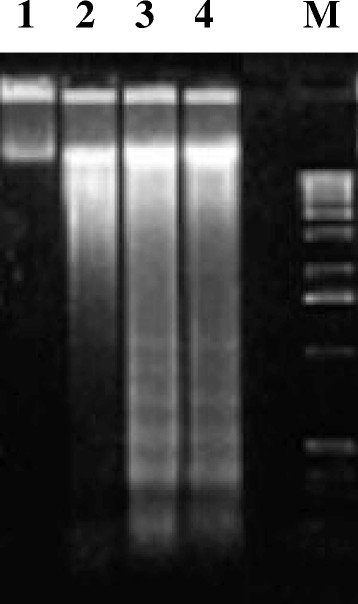
**PID15 induced apoptosis in isolated human peripheral lymphocytes.** Lymphocytes (1×10^6^ cells) ± PID-15 in 0.5 ml of HBSS, pH7.4, and reaction arrested in ice cooled bath. 2 μg of DNA from cells loaded on 0.8% agarose gel and electrophoresced. M. Hind III digest of λ-phage DNA molecular marker, 1.DNA isolated from untreated lymphocytes (1×10^6^ cells). 2. DNA isolated from NV-PLA_2_ (1 μg protein) treated lymphocytes. 3. As in 2 + PID-15 (0.1 μmoles). 4. As in 2 + PID-15 (0.2 μmoles).

Superoxide dismutase and Catalase did not have significant effect on the release of the PID-15, thus stating the non similarity to any clastogenic factors. However, Turmerin a potent 14 kDa antioxidant from Turmeric (*Curcuma longa* L.) showed significant inhibition of the secretion of PID15 indicating the involvement of free radicals other than superoxide and peroxide radicals in inducing DNA fragmentation. The DNA fragmentation of PID15 is shown in Figure 5. It induces apoptosis. The apoptosis inducing effect of PID15 was compared with NV-PLA_2_ thereby supporting the sequence data that PID15 act as apoptosis inducing factor.

### Sequence analysis of PID15

**Figure 6:**
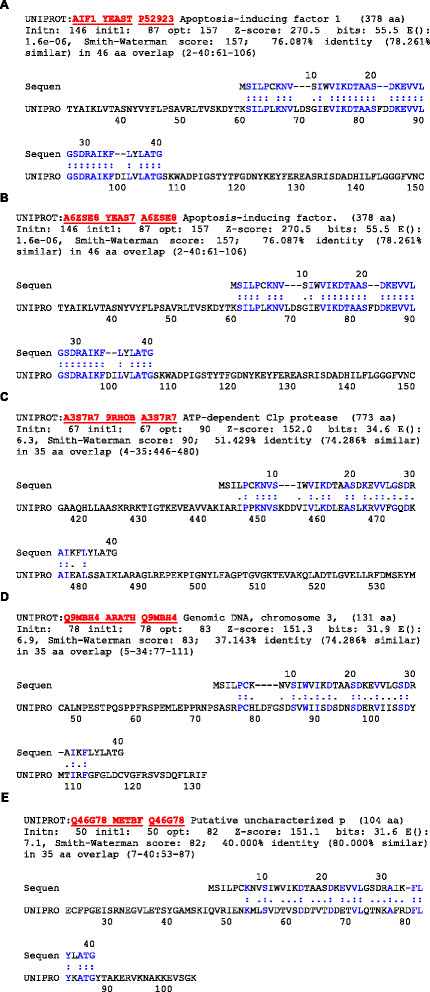
**ExPasy ProtParam sequence analysis of PID15.** Homology match for six different proteins such as **(A)** Apoptosis-inducing factor 1, **(B)** Apoptosis-inducing factor, **(C)** ATP-dependent Clp protease, **(D)** Genomic DNA, chromosome 3 and **(E)** Putative uncharacterized p. The similarity in homology to the above sequences in the database is highly significant, showing PID15 to be an Apoptosis Inducing factor.

PID15 sequence analysis gave 40 amino acids in the following order, *msilpcknvs iwvikdtaas dkevvlgsdr aikflylatg*. The homology search for the sequence revealed it to be an Apoptosis Inducing Factor (AIF). It showed a homology match for six different proteins such as Apoptosis-inducing factor 1, ATP-dependent Clp protease, Genomic DNA, chromosome 3 and Putative uncharacterized p (Figure 6). The similarity in homology to the above sequences in the database is highly significant. Apart from showing it to be an Apoptosis Inducing factor, PID-15 has sequence homology to an ATP dependent C1p protease, which refers it to act as protease in damaging the cellular structure. According to the ExPasy ProtParam tool for sequence analysis, the PID-15 was a stable protein with stability index of 12.33. The protein had an extinction coefficient of 6990, with absorbance (0.1%) of 1.605, assuming all cysteine residues appear to half cystines.

## Conclusions

The present investigation is novel of its kind demonstrating that human peripheral lymphocytes on exposure to NV-PLA_2_ secrete a novel apoptosis inducing factor, PID15 which further trigger the cell death process and act as a signaling molecule originating from cell membrane. However, a detailed investigation on the role played by this peptide during snake envenomations and its interaction with neighboring cells and also the changes associated with the intracellular environments needs much research.

## Competing interests

The authors declare that they have no competing interests.

## Authors’ contributions

MCK designed the whole experimental scheme, data interpretation and manuscript writing. LS contributed to the study concept. Both authors read and approved the final version of the manuscript.
